# Celecoxib combined with salirasib strongly inhibits pancreatic cancer cells in 2D and 3D cultures

**DOI:** 10.7150/ijms.47546

**Published:** 2020-07-09

**Authors:** Dongli Li, Yuran Ma, Wenfeng Liu, Xiang Ren, Min Chen, Xuetao Xu, Zhaojun Sheng, Kun Zhang, Renping Zhou, Susan Goodin, Xi Zheng

**Affiliations:** 1 School of Biotechnology and Health Sciences, Wuyi University, Jiangmen city, 529020, China.; 2 International Healthcare Innovation Institute (Jiangmen), Jiangmen city, Guangdong Province 529020, China.; 3 Department of Chemical Biology, Ernest Mario School of Pharmacy, Rutgers, The State University of New Jersey, Piscataway, NJ 08854, USA.; 4 Rutgers Cancer Institute of New Jersey, New Brunswick, NJ 08903, USA.

**Keywords:** Pancreatic cancer, 3D cultures, celecoxib, salirasib, NF-κB.

## Abstract

**Background/Aim:** Pancreatic adenocarcinoma is a highly malignant tumor. Synergistic combinations of anticancer agents for the effective treatment of pancreatic cancer patients are urgently needed. Here, we investigated the combined effect of celecoxib (CEL) and salirasib (SAL) on pancreatic cancer cells.

**Methods:** Cell viability and apoptosis were measured by the trypan blue assay, three-dimensional cultures, propidium iodide staining, and caspase-3 assay. NF-κB activation and the protein levels of Akt, pAkt, and Bcl-2 were determined by the luciferase reporter assay and western blot.

**Results:** Co-treatment with CEL and SAL had stronger effects on decreasing cell viability and inducing apoptosis in Panc-1 cells as compared with each agent individually. This combination strongly inhibited NF-κB activity and reduced pAkt and Bcl-2 levels in Panc-1 cells.

**Conclusion:** SAL in combination with CEL may represent a new approach for effective inhibition of pancreatic cancer.

## Introduction

Pancreatic adenocarcinoma is a highly aggressive cancer that was estimated to cause more than 45000 deaths in the United States in 2019 1. Pancreatic cancer arises from the morphologically and genetically distinct defined precursor lesions through a step-wise accumulation of genetic alterations. In the majority of the patients diagnosed with pancreatic cancer, symptoms do not develop until they are either unresectable or metastatic, rendering it difficult to cure 2, 3. Despite all advances in cancer treatment, it is still the fourth most frequent tumor-related cause of death in the Western world 1, 2. The 5-year survival rate for pancreatic cancer is less than 5%, and conventional treatment approaches, such as surgery, radiation, chemotherapy, or combinations thereof, have had little impact on the course of this aggressive neoplasm. A median survival of <6 months has remained unchanged for the last three decades 3, 4, 5. Almost all patients with pancreatic cancer develop metastases resulting in death because of the debilitating metabolic effects of its unrestrained growth. The low survival rate of patients indicates an increased need for novel anticancer agents and effective combination therapies for the treatment of pancreatic cancer.

**Figure 1:**
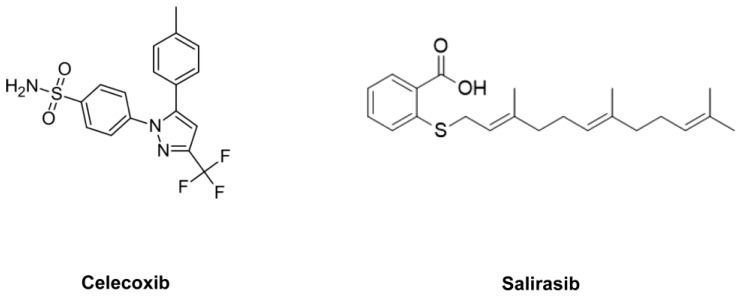
Structures of SAL and CEL.

Ras is a family of genes involved in regulating cell proliferation, differentiation, adhesion and apoptosis. It has been reported that KRAS mutations occur in more than 90% of pancreatic cancers 6. This mutation results in constitutive activation of the protein, which no longer requires a ligand for activation 7. S-*trans, trans-*farnesylthiosalicylic acid (FTS; also known as Salirasib [SAL]; Fig. 1), a synthetic Ras inhibitor, SAL acts as a functional Ras antagonist in cells that affects Ras-membrane interactions by dislodging the protein from its anchorage domains, which facilitates its degradation and thus reduces the cellular active Ras content 8, 9. SAL has been shown to inhibit the growth of H-Ras-, K-Ras-, and N-Ras-transformed rodent fibroblasts *in vitro* and inhibit the anchorage-independent growth of several cancer cell lines 10, 11, 12, 13.

It has been reported that overexpression of cyclooxygenase-2 (COX-2) is present in the majority of pancreatic cancer patients and is closely related to the development of pancreatic cancers 14, 15, 16. Thus, COX-2 inhibitors such as celecoxib (CEL) have the potential to suppress their invasion and metastasis. COX-2 overexpression results in increased production of prostaglandins and Akt activation 17. One approach for inhibiting pancreatic cancer growth and progression is the simultaneous use of a COX-2 inhibitor such as CEL (Fig. 1) with a Ras inhibitor such as SAL. This combination may synergistically suppress pancreatic cancer growth and stimulate apoptosis.

The present study was designed to explore the effects of CEL alone or in combination with SAL on the growth and apoptosis of pancreatic cancer Panc-1 cells. We determined the effects of CEL and SAL indiviadually or in combination on pancreatic cancer cells in conventional monolayer cultures and in three-dimensional (3D) cultures. Our study provides the first evidence that the combination of CEL and SAL strongly inhibited growth and induced apoptosis in Panc-1 cells, and the effects of this combination were also associated with the inhibition of NF-κB activity and decreased levels of Bcl-2 and phospho-Akt in Panc-1 cells.

## Materials and Methods

### Cells and reagents

Panc-1 cells were obtained from the American Type Culture Collection (ATCC, Rockville, MD, USA). CEL was purchased from LC Laboratories (Woburn, MA, USA). Matrigel was obtained from BD Biosciences (Bedford, MA, USA). SAL was obtained from Sigma-Aldrich (St. Louis, MO, USA). Dulbecco's modified Eagle's medium (DMEM), penicillin-streptomycin, L-glutamine, and fetal bovine serum (FBS) were obtained from Gibco (Grand Island, NY, USA). Panc-1 cells were maintained in DMEM containing 10% FBS and supplemented with penicillin (100 units/mL)-streptomycin (100 µg/mL) and L-glutamine (300 µg/mL). Cultured cells were grown at 37 °C in a humidified atmosphere of 50 mL/L CO_2_ and passaged twice a week.

### Determination of viable cell numbers

The number of viable cells after each treatment was determined using a hemocytometer under a light microscope (Nikon Optiphot, Tokyo, Japan). Cell viability was determined by the trypan blue exclusion assay, which was performed by mixing 80 µL of cell suspension with 20 µL of 0.04 g/mL trypan blue solution for 2 min. Blue cells were counted as dead cells, whereas undyed cells were counted as live cells.

### Assessment of apoptotic cells by morphology and activation of caspase-3

Apoptosis was determined by morphological assessment of cells stained with propidium iodide. Briefly, cytospin slides were prepared after each experiment, and cells were fixed with acetone/methanol (1:1) for 10 min at room temperature, stained with propidium iodide (1 µg/mL in PBS) for 10 min, and analyzed using a fluorescence microscope (Nikon Eclipse TE200, Tokyo, Japan). Apoptotic cells were identified by classical morphological features, including nuclear condensation, cell shrinkage, and formation of apoptotic bodies 18. Caspase-3 activation was measured using an EnzoLyte AMC Caspase-3 Assay Fluorimetric Kit (AnaSpec, Fremont, CA, USA) following the manufacturer's instructions 19. Briefly, 1 × 10^5^ cells were plated in triplicate in a flat-bottomed 96-well plate. After treatment with SAL and/or CEL, caspase-3 substrate was added to each well. Plates were incubated for 30 min at room temperature. Fluorescence intensity was measured using a Tecan Inifinite M200 plate reader (Tecan US Inc., Durham, NC, USA).

### NF-κB-dependent reporter gene expression assay

NF-κB transcriptional activity was measured by the NF-κB-luciferase reporter gene expression assay 20. The NF-κB-luciferase construct was transiently transfected into Panc-1 cells using Lipofectamine 2000 (Invitrogen Life Tech, Grand Island, NY, USA) following the manufacturer's instructions. The cells were then treated with CEL or SAL alone or in combination for 24 h, and the NF-κB-luciferase activities were measured using luciferase assay kits (E1500, Promega, Madison, WI, USA) according to the manufacturer' s instructions.

### Three-dimensional (3D) cell culture

Panc-1 cells were mixed with Matrigel (Collaborative Research, Bedford, MA, USA) on ice at a density of 0.5 × 10^5^ cells/mL. Matrigel containing Panc-1 cells was placed in a 12-well plate (1 mL/well) and incubated at 37 °C for 2 h to allow the Matrigel to solidify. Subsequently, DMEM was added to each well on top of the gel. The cells were incubated for 24 h and then treated with CEL or SAL alone or in combination once every other day. On day 10, the 3D cultures were examined under a microscope (Nikon Optiphot, Tokyo, Japan) for the formation of tissue-like structures.

### Western blotting

At the end of the experiment, cell lysates were prepared as described earlier. Proteins were subjected to sodium dodecyl sulfate polyacrylamide gel electrophoresis and transferred to nitrocellulose membranes. After blocking nonspecific binding sites with blocking buffer, the membrane was incubated overnight at 4 °C with Akt (#9271, Cell Signaling Technology, Beverly, MA, USA) and Bcl-2 (#15071, Cell Signaling Technology) primary antibodies, and β-actin (#4970, Cell Signaling Technology) was used as a loading control. Following removal of the primary antibody, the membrane was washed three times with TBS (PBS containing 0.5 mL/L Tween 20) at room temperature and then incubated with fluorochrome-conjugated secondary antibody (Santa Cruz Biotechnology Inc., CA, USA). The membrane was then washed with TBS three times. Final detection was performed with an Odyssey infrared imaging system (Li-Cor Biotechnology, Lincoln, NE, USA).

### Statistical analyses

Analysis of synergy was performed using CompuSyn software. The combination index (CI)-fraction affected (CI-Fa) curve demonstrates the relationship between the CI value and the effective level of a certain biological indicator. If CI < 1, then the compounds are thought to have synergistic effects. If CI > 1 or = 1, then the compounds are thought to be antagonistic or additive, respectively. Analysis of variance (ANOVA) with the Tukey-Kramer test was used to compare results among different experimental groups.

## Results

### Effects of SAL and CEL on cell viability and apoptosis of pancreatic cancer Panc-1 cells

**Figure 2:**
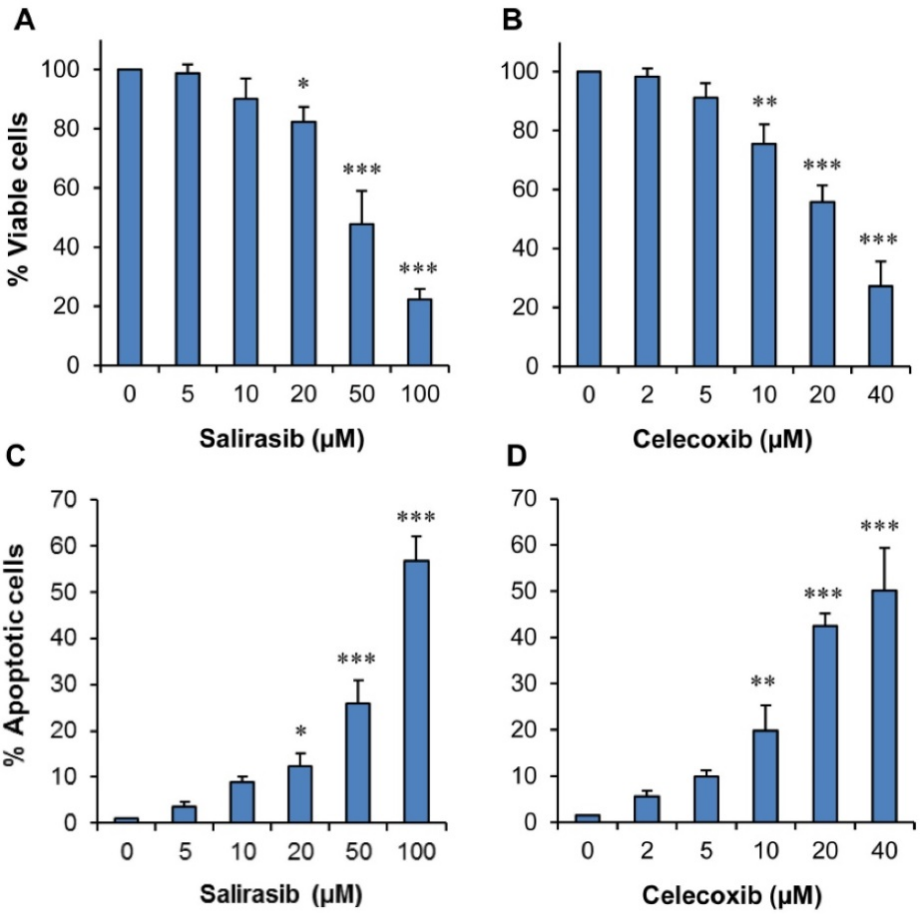
** Effects of SAL or CEL on Panc-1 cell growth and apoptosis.** Panc-1 cells were seeded in cell culture dishes with DMEM, incubated for 24 h, and then treated with SAL (5-100 µM) or CEL (2-40 µM) for 72 h. The viable cell number was determined by the trypan blue exclusion assay, and apoptotic cells were determined by propidium iodide staining and morphological assessment. (A) Percent of viable cells in the SAL-treated group. (B) Percent of viable cells in the CEL-treated group. (C) Percent of apoptosis in the SAL-treated group. (D) Percent of apoptosis in the CEL-treated group. Data are expressed as the mean ± SD from three separate experiments. *P<0.05 *vs.* control; **P<0.01 *vs.* control; ***P<0.001 *vs.* control.

The effects of SAL or CEL on pancreatic cancer cell viability and apoptosis were examined using pancreatic cancer Panc-1 cells. In these experiments, Panc-1 cells were treated with SAL or CEL for 72 h, and the viable cell number following treatment was determined by the trypan blue exclusion assay. As shown in Figure 2A&B, treatment of the cells with SAL (5-100 µM) or CEL (2-40 µM) resulted in a concentration-dependent decrease in the number of viable cells. Moreover, SAL or CEL dose-dependently induced apoptosis in Panc-1 cells (Figure 2C&D).

**Figure 3:**
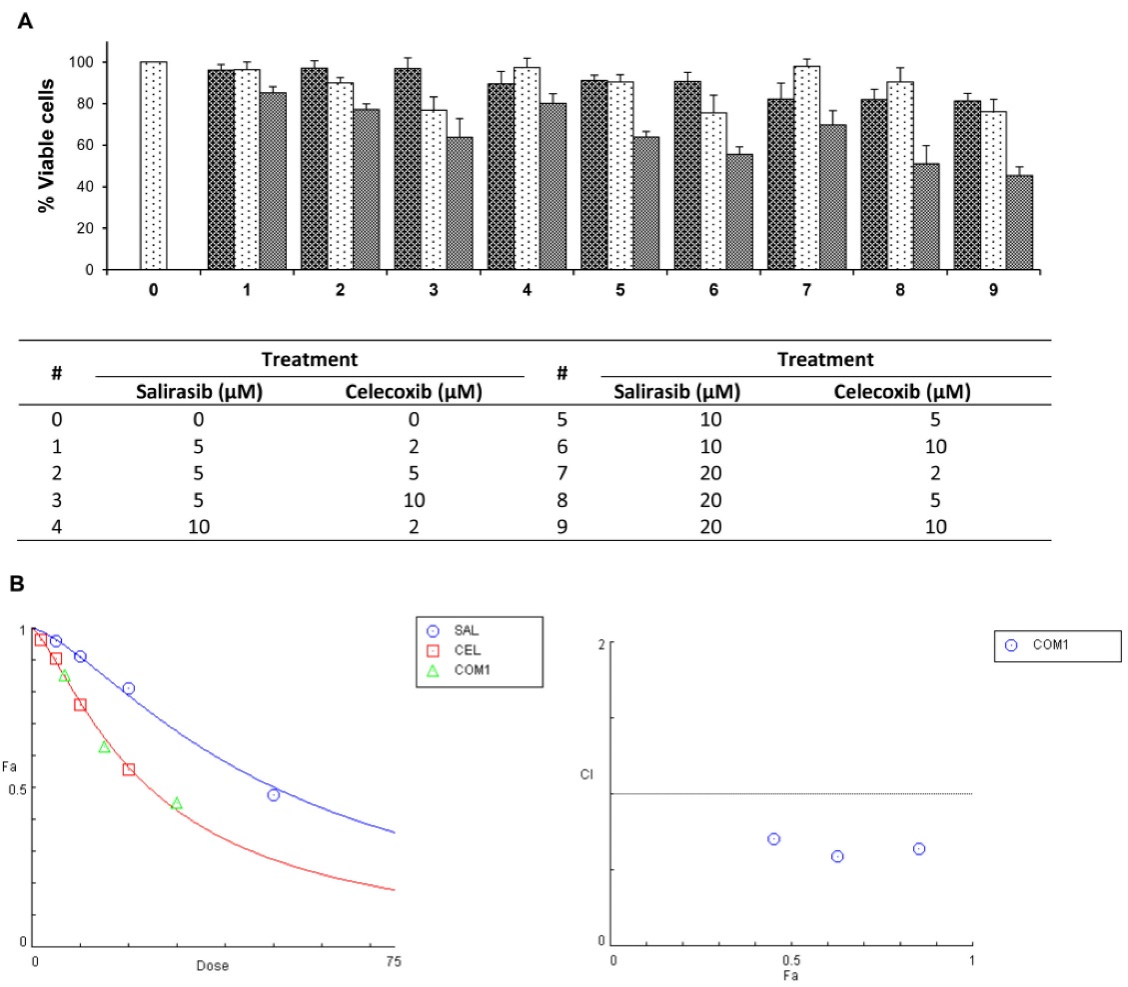
** Effects of combinations of SAL and CEL at different ratios on Panc-1 cell growth.** Panc-1 cells were seeded in cell culture dishes with DMEM, incubated for 24 h, and then treated with SAL (5, 10, and 20 µM) and CEL (2, 5, and 10 µM) alone or in combination for 72 h. The viable cell number was determined by the trypan blue exclusion assay. Statistical analysis for synergy was performed using CompuSyn software. (A) Number of viable cells. (B) left, dose-fraction affected (Dose-Fa); right, combination index-fraction affected (CI-Fa). Data are expressed as the mean ± SD from three separate experiments.

Combinations of SAL and CEL at different ratios were found to more potently inhibit Panc-1 cell viability than either agent individually (Figure 3A). Statistical analysis based on an isobologram showed that SAL and CEL in combination synergistically decreased the viability of Panc-1 cells (Figure 3B). The CI for the combination of SAL and CEL at 5 µM + 2 µM, respectively, was calculated as 0.64, that at 10 µM + 5 µM was 0.59, and that at 20 µM + 10 µM was 0.71, indicating a synergistic effect between SAL and CEL.

**Figure 4:**
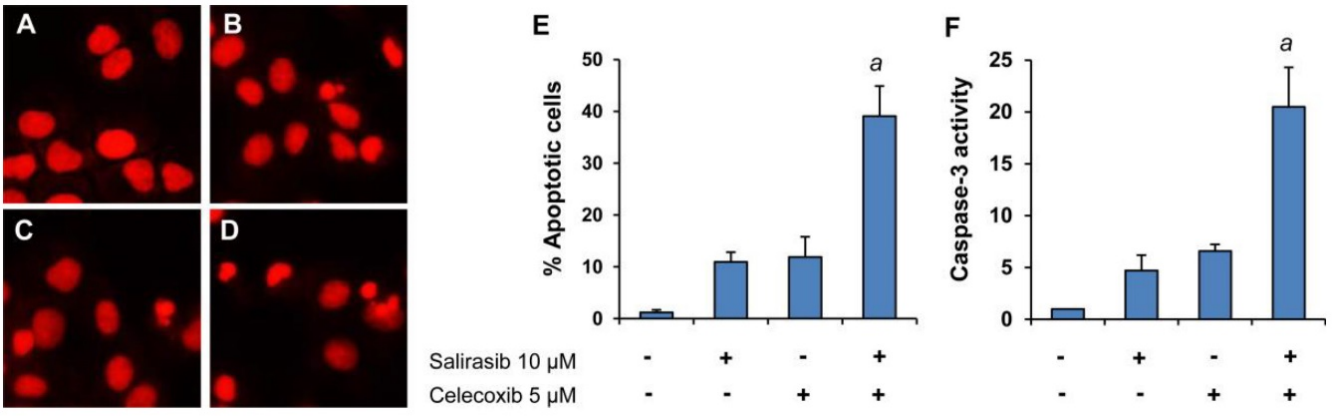
** Effects of SAL alone or in combination with CEL on apoptosis of Panc-1 cells.** Panc-1 cells were seeded in cell culture dishes with DMEM, incubated for 24 h, and then treated with SAL (10 µM) and CEL (5 µM) alone or in combination for 72 h. The number of apoptotic cells was determined by propidium iodide staining and caspase-3 assay. (A-D) Representative micrographs of propidium iodide staining. (A) Control cells. (B) Cells treated with SAL. (C) Cells treated with CEL. (D) Cells treated with a combination of SAL and CEL. (E) Percent apoptosis determined by propidium iodide staining. (F) Fold increase in caspase-3 activity. Each value represents the mean ± SD from three separate experiments. ^a^*P*<0.001 *vs.* treatment with SAL or CEL alone.

We further determined the effect of the combination on apoptosis in Panc-1 cells by propidium iodide staining and caspase-3 assay. As shown in Figure 4, treatment with SAL and CEL in combination resulted in stronger induction of apoptosis than either drug individually. Figure 4A-D displays representative micrographs of propidium iodide staining in Panc-1 cells from the control group (A), SAL-treated group (B), CEL-treated group (C), and combination-treated group (D). The percentages of apoptotic cells in different treatment groups are shown in Figure 4E. ANOVA revealed a significantly higher percentage of apoptosis in the combination group than that in the SAL- or CEL-treated groups (*P*<0.001). Apoptosis in Panc-1 cells treated with SAL or CEL alone or in combination were also determined using the caspase-3 assay. As shown in Figure 4F, the combination of SAL and CEL had a stronger effect on increasing caspase-3 activity than either drug alone. Moreover, ANOVA revealed that the caspase-3 activity was significantly higher in the combination group than that in the SAL- or CEL-treated groups (*P*<0.001).

### Effects of SAL and CEL on Panc-1 cells in 3D cultures

**Figure 5:**
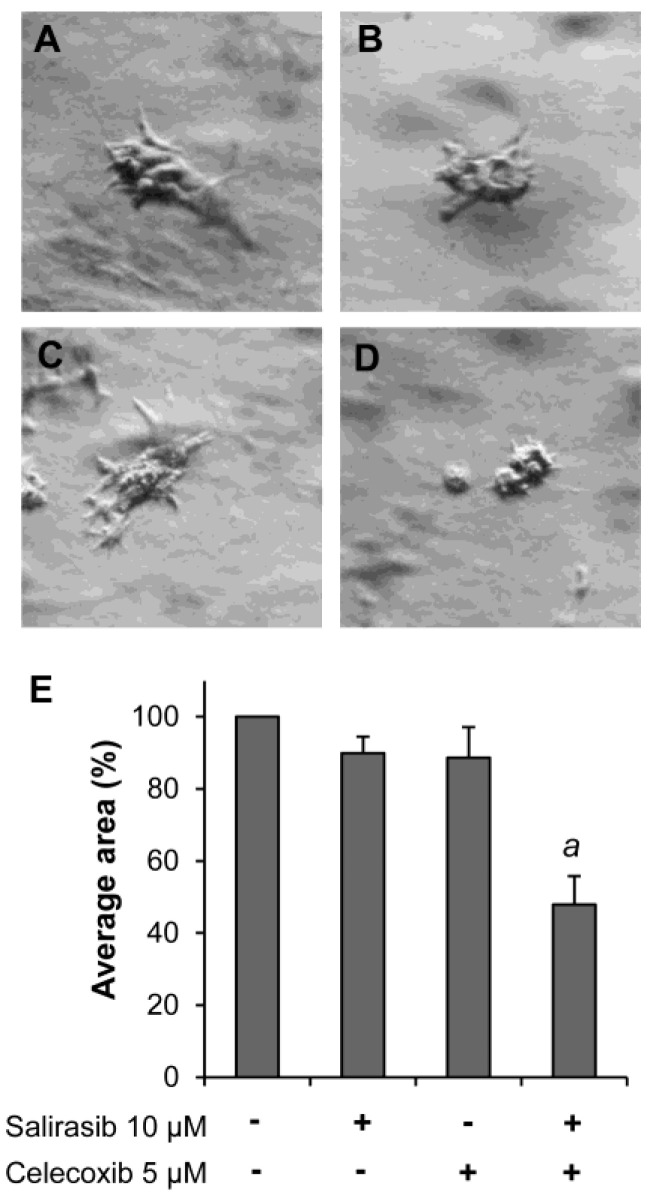
** Effect of SAL and/or CEL on Panc-1 cells in 3D cultures.** Panc-1 cells were seeded in Matrigel in a 12-well plate (1 mL/well) and treated with SAL and/or CEL once every other day for 10 days. Representative micrographs of Panc-1 3D cell cultures of control cells (A), SAL-treated (B), CEL-treated (C), and combination-treated (D) groups are shown. Data are expressed as the mean ± SD from three separate experiments. ^a^*P*<0.001 *vs.* treatment with SAL or CEL alone.

A 3D cell culture model was used to determine the effects of CEL and SAL alone or in combination on the formation and growth of 3D tissue-like structures. As shown in Figure 5A, Panc-1 cells formed a tissue-like morphology in 3D culture in the extracellular matrix gel. Treatment with SAL or CEL individually had an inhibitory effect on the formation and growth of the tissue-like structures (Fig. 5B and C), whereas the drugs in combination more potently inhibited the formation of these structures (Fig. 5D). The average area of the tissue-like structure was measured using Image J software. As shown in Figure 5E, treatment with the combination of SAL and CEL had a more potent effect on decreasing the size of the tissue-like structure than either drug alone (*P*<0.001).

### Inhibition of NF-κB activity and expression of Bcl-2 and pAkt in Panc-1 cells treated with SAL and/or CEL

**Figure 6:**
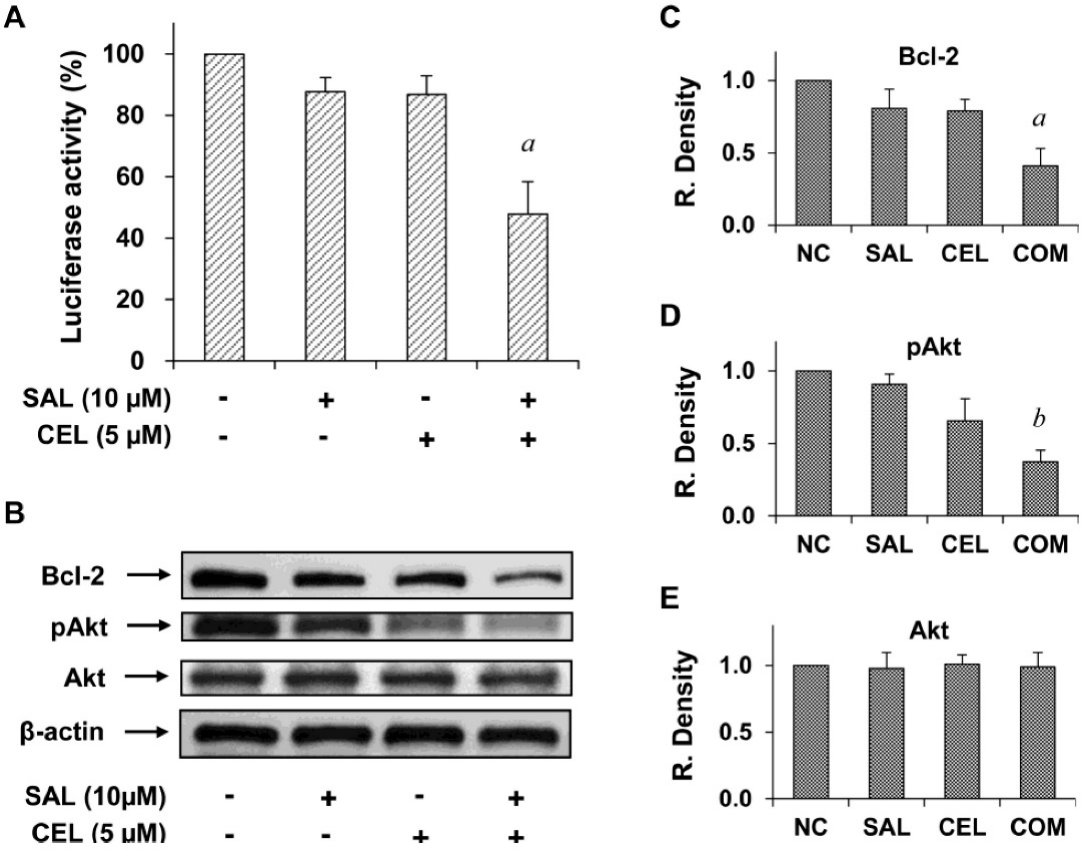
** Effects of SAL and/or CEL on NF-**κ**B transcriptional activity and the levels of Bcl-2 and Akt in Panc-1 cells.** (A) Panc-1 cells were transfected with an NF-κB-luciferase construct using Lipofectamine 2000 (LF2000, Invitrogen Life Technology). The cells were treated with SAL alone or in combination with CEL for 24 h. Luciferase activity was determined using a luciferase assay kit (E1500, Promega). Each value represents the mean ± S.E. from three separate experiments. (B) Panc-1 cells were treated with SAL alone or in combination with CEL for 24 h. The levels of Bcl-2, Akt, and phospho-Akt (pAkt) were determined by western blot analysis. Data are expressed as the mean ± SD from three separate experiments. ^a^*P*<0.01 *vs.* treatment with SAL or CEL alone. ^b^*P*<0.001 *vs.* treatment with SAL alone, and *P*<0.05 *vs.* treatment with CEL alone.

To investigate the mechanisms of action of SAL and CEL, we first determined the effect of these two drugs on NF-κB, which was found to play an important role in pancreatic cancer. Treating cells with SAL and CEL in combination had a stronger inhibitory effect on NF-κB activity than either drug alone (Figure 6A). NF-κB activity was significantly lower in the combination group than in the SAL- or CEL-treated groups (*P*<0.01), as analyzed by ANOVA. We further examined the effect of SAL and CEL on the level of Bcl-2, an anti-apoptotic protein regulated by NF-κB. As shown in Figure 6B, treatment with SAL and CEL in combination had a stronger effect than either drug alone on decreasing the level of Bcl-2 in Panc-1 cells. In addition, cellular levels of phospho-Akt (pAkt) and Akt were determined and found to be strongly decreased by the combination of SAL and CEL in Panc-1 cells (Figure 6B). The levels of Bcl-2, pAkt and Akt relative to control as determined by band density measurement are shown in Figure 6C-E. The level of Bcl-2 in the combination-treated group was significantly lower than that in the SAL- and CEL-treated groups (*P*<0.01). The level of pAkt in the combination-treated group was significantly lower than that in the SAL- (*P*<0.001) and CEL-treated groups (*P*<0.05). The levels of Akt did not change after treatment with SAL and CEL.

## Discussion

In the present study, we demonstrated that SAL in combination with CEL synergistically decreased the viability of pancreatic cancer Panc-1 cells. This drug combination was found to have more potent effect on inducing apoptosis in Panc-1 cells than either drug used individually. To the best of our knowledge, this is the first study indicating strong combined effects of SAL and CEL on viability and apoptosis in human pancreatic cancer cells.

The inhibitory effect of SAL and CEL alone or in combination on Panc-1 cells in 3D cultures was investigated in the present study. Compared to conventional 2D monolayer cell cultures, the 3D culture system mimics the structural architecture and differentiation ability of the tumor tissues. It is well known that cell-cell and cell-matrix interactions within the 3D microenvironment are important for the physiological function and response of cancer cells to anticancer agents 21, 22. In the present study, we found that Panc-1 cells formed 3D tissue-like morphology in Matrigel which contained an extra cellular matrix. Treatment of Panc-1 cells with SAL and CEL in combination had a stronger inhibitory effect than either drug alone on the formation of tissue-like morphology in 3D cultures.

Ras signaling is a major junction of various signaling pathways. For this reason, Ras and its effectors serve as important targets for therapeutic intervention in pancreatic cancer 23. SAL is a synthetic Ras inhibitor that affects Ras-membrane interactions and has been shown to effectively inhibit the growth of human cancer cells 10, 11, 12, 13. In the present study, we found that SAL dose-dependently decreased the viability and stimulated apoptosis of pancreatic cancer Panc-1 cells as well as inhibited the formation and growth of tumor tissue-like structures in 3D cell cultures. It was previously shown that KRAS activates NF-κB, which is crucial for cell survival and tumorigenesis 24, 25. Here, we found that SAL inhibited NF-κB, as determined by the luciferase reporter assay. Moreover, SAL decreased the level of NF-κB downstream of the anti-apoptotic protein Bcl-2. SAL in combination with CEL had a more potent effect on inhibiting NF-κB activity and decreasing the Bcl-2 level than either drug used individually. These results indicated that inhibition of the NF-κB signaling pathway may play a role in the inhibitory effect of SAL and CEL in pancreatic cancer cells.

CEL, a selective COX-2 inhibitor, has been shown previously to inhibit the growth of human pancreatic cancer cell lines 26, 27. A previous study demonstrated that CEL inhibited angiogenesis, tumor growth, and metastasis in pancreatic xenograft tumors 28. In a study using a COX-2-overexpressing mouse model, CEL was shown to inhibit pancreatic cancer formation 29. Here, we found that CEL dose-dependently decreased viability and stimulated apoptosis in Panc-1 cells as well as inhibited NF-κB activation and decreased the level of NF-κB downstream of the anti-apoptotic protein Bcl-2. The NF-κB signaling pathway has been shown to play an important role in pancreatic cancer development, metastasis, and chemoresistance 30, 31, 32; furthermore, this pathway is considered as a novel target in pancreatic cancer 33. Our results demonstrated that CEL in combination with SAL potently inhibited NF-κB activity. The mechanisms for the regulation of NF-κB activity by the combination of SAL and CEL are not clear. KRAS has been shown to activate NF-κB 24, 34. SAL is a RAS inhibitor and the inhibitory effect of SAL on NF-κB activation found in the present study may be mediated by inhibition of RAS activity. The COX-2 enzyme catalyzes the production of prostaglandin (PGE_2_) which can activate NF-κB 27. CEL inhibits COX-2 and decreases the production of PGE_2_ leading to suppression of NF-κB activation. SAL and CEL target different pathways involved in the regulation of NF-κB and the combination of these two drugs resulted in potent suppression of NF-κB. Our studies suggest that simultaneous inhibition of the RAS pathway and the COX-2/PGE_2_ axis may represent an effective strategy for suppression of NF-κB activity. The effective inhibition of NF-κB pathway by the combination of SAL and CEL was further confirmed by a strong decrease in the level of Bcl-2 which is an anti-apoptotic protein and a downstream target of NF-κB. In addition, our study revealed that pAkt levels were decreased by CEL and strongly decreased by co-treatment with CEL and SAL. It has been well documented that PTEN/Akt signaling is one of the major players in cancer biology and affects a wide range of cancer cell behaviors, including cell viability, senescence, proliferation, migration, and invasion, by regulating the activities of various transcription factors and signaling molecules 35. Suitable combinations that target multiple signaling pathways may increase the effectiveness of anticancer agents in pancreatic cancer. SAL and CEL strongly inhibited NF-κB and Akt in pancreatic cancer cells. Therefore, the combination of these two drugs may be an effective approach for inhibiting pancreatic cancer.

In summary, we demonstrated that SAL in combination with CEL synergistically decreased the viability of human pancreatic cancer Panc-1 cells. The drug combination also strongly inhibited Panc-1 cells in 3D cell cultures. The strong effects of SAL in combination with CEL on pancreatic cancer cells were associated with decreases in NF-κB activity and Bcl-2 and pAkt levels. Our results indicate that combining SAL and CEL may be an effective approach to inhibit pancreatic cancer cells, and further studies with suitable animal models to determine the *in vivo* effects of this drug combination are warranted.

## References

[1] Siegel RL, Miller KD, Jemal A (2019). Cancer statistics, 2019. CA Cancer J Clin.

[2] Zavoral M, Minarikova P, Zavada F (2011). Molecular biology of pancreatic cancer. World journal of gastroenterology.

[3] Li D, Xie K, Wolff R (2004). Pancreatic cancer. The Lancet.

[4] Sultana A, Tudur Smith C, Cunningham D (2008). Meta-analyses of chemotherapy for locally advanced and metastatic pancreatic cancer: results of secondary end points analyses. Br J Cancer.

[5] Zalatnai A (2003). Pancreatic Cancer - a Continuing Challenge in Oncology. Pathol Oncol Res.

[6] Jonckheere N, Vasseur R, Van Seuningen I (2017). The cornerstone K-RAS mutation in pancreatic adenocarcinoma: From cell signaling network, target genes, biological processes to therapeutic targeting. Crit Rev Oncol Hematol.

[7] Watanabe M, Nobuta A, Tanaka J (1996). An effect of K-ras gene mutation on epidermal growth factor receptor signal transduction in PANC-1 pancreatic carcinoma cells. Int J Cancer.

[8] Aharonson Z, Gana-Weisz M, Varsano T (1998). Stringent structural requirements for anti-Ras activity of S-prenyl analogues. Biochim Biophys Acta.

[9] Haklai R, Weisz MG, Elad G (1998). Dislodgment and accelerated degradation of Ras. Biochemistry.

[10] Elad G, Paz A, Haklai R (1999). Targeting of K-Ras 4B by S-trans,trans-farnesyl thiosalicylic acid. Biochimica et Biophysica Acta (BBA) - Molecular Cell Research.

[11] Marom M, Haklai R, Ben-Baruch G (1995). Selective inhibition of Ras-dependent cell growth by farnesylthiosalisylic acid. J Biol Chem.

[12] McPherson RA, Conaway MC, Gregory CW (2004). The novel Ras antagonist, farnesylthiosalicylate, suppresses growth of prostate cancer *in vitro*. Prostate.

[13] Erlich S, Tal-Or P, Liebling R (2006). Ras inhibition results in growth arrest and death of androgen-dependent and androgen-independent prostate cancer cells. Biochem Pharmacol.

[14] Hillion J, Smail SS, Di Cello F (2012). The HMGA1-COX-2 axis: a key molecular pathway and potential target in pancreatic adenocarcinoma. Pancreatology.

[15] Kokawa A, Kondo H, Gotoda T (2001). Increased expression of cyclooxygenase-2 in human pancreatic neoplasms and potential for chemoprevention by cyclooxygenase inhibitors. Cancer.

[16] Tucker ON, Dannenberg AJ, Yang EK (1999). Cyclooxygenase-2 Expression Is Up-Regulated in Human Pancreatic Cancer. Cancer Res.

[17] Krysan K, Reckamp KL, Dalwadi H (2005). Prostaglandin E2 activates mitogen-activated protein kinase/Erk pathway signaling and cell proliferation in non-small cell lung cancer cells in an epidermal growth factor receptor-independent manner. Cancer Res.

[18] Chen X, Liu Y, Wu J (2016). Mechanistic Study of Inhibitory Effects of Atorvastatin and Docetaxel in Combination on Prostate Cancer. Cancer Genomics Proteomics.

[19] Wei X, Du ZY, Cui XX (2012). Effects of cyclohexanone analogues of curcumin on growth, apoptosis and NF-kappaB activity in PC-3 human prostate cancer cells. Oncol Lett.

[20] Zheng X, Cui XX, Gao Z (2012). Effects of 12-O-tetradecanoylphorbol-13-acetate in combination with gemcitabine on Panc-1 pancreatic cancer cells cultured *in vitro* or Panc-1 tumors grown in immunodeficient mice. Int J Oncol.

[21] Tsunoda T, Ishikura S, Doi K (2014). Resveratrol induces luminal apoptosis of human colorectal cancer HCT116 cells in three-dimensional culture. Anticancer Res.

[22] Akeda K, Nishimura A, Satonaka H (2009). Three-dimensional alginate spheroid culture system of murine osteosarcoma. Oncol Rep.

[23] Bisht S, Feldmann G (2018). Novel Targets in Pancreatic Cancer Therapy - Current Status and Ongoing Translational Efforts. Oncol Res Treat.

[24] Yang L, Zhou Y, Li Y (2015). Mutations of p53 and KRAS activate NF-kappaB to promote chemoresistance and tumorigenesis via dysregulation of cell cycle and suppression of apoptosis in lung cancer cells. Cancer Lett.

[25] Chenette EJ (2009). Cancer: A Ras and NF-kappaB pas de deux. Nat Rev Drug Discov.

[26] Ding XZ, Hennig R, Adrian TE (2003). Lipoxygenase and cyclooxygenase metabolism: new insights in treatment and chemoprevention of pancreatic cancer. Mol Cancer.

[27] El-Rayes BF, Ali S, Sarkar FH (2004). Cyclooxygenase-2-dependent and -independent effects of celecoxib in pancreatic cancer cell lines. Mol Cancer Ther.

[28] Raut CP, Nawrocki S, Lashinger LM (2004). Celecoxib inhibits angiogenesis by inducing endothelial cell apoptosis in human pancreatic tumor xenografts. Cancer Biol Ther.

[29] Wei D, Wang L, He Y (2004). Celecoxib inhibits vascular endothelial growth factor expression in and reduces angiogenesis and metastasis of human pancreatic cancer via suppression of Sp1 transcription factor activity. Cancer Res.

[30] Garcea G, Dennison AR, Steward WP (2005). Role of inflammation in pancreatic carcinogenesis and the implications for future therapy. Pancreatology.

[31] Yuan P, He XH, Rong YF (2017). KRAS/NF-kappaB/YY1/miR-489 Signaling Axis Controls Pancreatic Cancer Metastasis. Cancer Res.

[32] Li Q, Yang G, Feng M (2018). NF-kappaB in pancreatic cancer: Its key role in chemoresistance. Cancer Lett.

[33] Pramanik KC, Makena MR, Bhowmick K (2018). Advancement of NF-kappaB Signaling Pathway: A Novel Target in Pancreatic Cancer. Int J Mol Sci.

[34] Bera A, Zhao S, Cao L (2013). Oncogenic K-Ras and loss of Smad4 mediate invasion by activating an EGFR/NF-kappaB Axis that induces expression of MMP9 and uPA in human pancreas progenitor cells. PLoS One.

[35] Garrett JT, Chakrabarty A, Arteaga CL (2011). Will PI3K pathway inhibitors be effective as single agents in patients with cancer?. Oncotarget.

